# Validation of questionnaire-reported hearing with medical records: A report from the Swiss Childhood Cancer Survivor Study

**DOI:** 10.1371/journal.pone.0174479

**Published:** 2017-03-23

**Authors:** Annette Weiss, Grit Sommer, Rahel Kuonen, Katrin Scheinemann, Michael Grotzer, Martin Kompis, Claudia E. Kuehni, on behalf of the Swiss Paediatric Oncology Group (SPOG)

**Affiliations:** 1 Swiss Childhood Cancer Registry, Institute of Social and Preventive Medicine, University of Bern, Bern, Switzerland; 2 Division of Pediatric Hematology/ Oncology, University Children`s Hospital Basel, Basel, Switzerland; 3 Department of Pediatric Oncology, University Children`s Hospital Zurich, University of Zurich, Zurich, Switzerland; 4 Department of ENT, Head and Neck Surgery, University Hospital Bern, University of Bern, Bern, Switzerland; 5 Children’s University Hospital of Bern, University of Bern, Bern, Switzerland; Universidad de Navarra, SPAIN

## Abstract

**Background:**

Hearing loss is a potential late effect after childhood cancer. Questionnaires are often used to assess hearing in large cohorts of childhood cancer survivors and it is important to know if they can provide valid measures of hearing loss. We therefore assessed agreement and validity of questionnaire-reported hearing in childhood cancer survivors using medical records as reference.

**Procedure:**

In this validation study, we studied 361 survivors of childhood cancer from the Swiss Childhood Cancer Survivor Study (SCCSS) who had been diagnosed after 1989 and had been exposed to ototoxic cancer treatment. Questionnaire-reported hearing was compared to the information in medical records. Hearing loss was defined as ≥ grade 1 according to the SIOP Boston Ototoxicity Scale. We assessed agreement and validity of questionnaire-reported hearing overall and stratified by questionnaire respondents (survivor or parent), sociodemographic characteristics, time between follow-up and questionnaire and severity of hearing loss.

**Results:**

Questionnaire reports agreed with medical records in 85% of respondents (kappa 0.62), normal hearing was correctly assessed in 92% of those with normal hearing (n = 249), and hearing loss was correctly assessed in 69% of those with hearing loss (n = 112). Sensitivity of the questionnaires was 92%, 74%, and 39% for assessment of severe, moderate and mild bilateral hearing loss; and 50%, 33% and 10% for severe, moderate and mild unilateral hearing loss, respectively. Results did not differ by sociodemographic characteristics of the respondents, and survivor- and parent-reports were equally valid.

**Conclusions:**

Questionnaires are a useful tool to assess hearing in large cohorts of childhood cancer survivors, but underestimate mild and unilateral hearing loss. Further research should investigate whether the addition of questions with higher sensitivity for mild degrees of hearing loss could improve the results.

## Introduction

Hearing loss is a late effect of childhood cancer that is especially common after ototoxic treatments such as platinum chemotherapy, cranial radiation, and brain surgery [1–3]. Audiometric testing is the best way to assess hearing loss, but particularly for the purpose of research comprehensive audiometric testing in large cohorts of childhood cancer survivors is not always feasible. Questionnaires are often used instead to ask about survivors’ hearing [2,4–6].

Previous studies of the validity of questionnaire-reported hearing have been conducted mainly in older populations [7–10]. Childhood cancer survivors are different. First, they may have had several hearing tests during cancer treatment and long-term follow-up. After completion of cranial radiation therapy, a yearly audiological evaluation for five years is recommended for survivors. After completion of platinum chemotherapy, a baseline evaluation at entry into follow-up is recommended, followed by yearly testing if hearing loss is detected [11]. Second, survivors can develop hearing loss very early in life and questionnaires for young survivors are often directed to their parents [5,12]. It is important to know if validity depends on the person who answered the questionnaire (survivor or parent), and if sociodemographic and clinical characteristics of survivors also play a role. However, no study has yet examined the validity of questionnaire-reported hearing in childhood cancer survivors.

We therefore assessed validity and agreement of questionnaire-reported hearing in childhood cancer survivors using medical records as reference, and determined factors associated with agreement including the person answering the questionnaire (survivor or parent), sociodemographic characteristics, time between follow-up and questionnaire, and levels of hearing loss.

## Methods

### Study population

The Swiss Childhood Cancer Survivor Study (SCCSS) is a population-based, long-term cohort study of all patients registered in the Swiss Childhood Cancer Registry (SCCR) who were diagnosed between 1976 and 2005 at age ≤ 20 years, and survived ≥5 years after initial diagnosis of cancer [12]. The SCCR is a population-based registry that includes all children and adolescents in Switzerland diagnosed with leukemia, lymphoma, central nervous system (CNS) tumors, malignant solid tumors, or Langerhans cell histiocytosis before the age of 21 [13]. Ethics approval was granted through the Ethics Committee of the Canton of Bern to the SCCR and SCCSS (KEK-BE: 166/2014).

### Inclusion criteria

**Fig 1:**
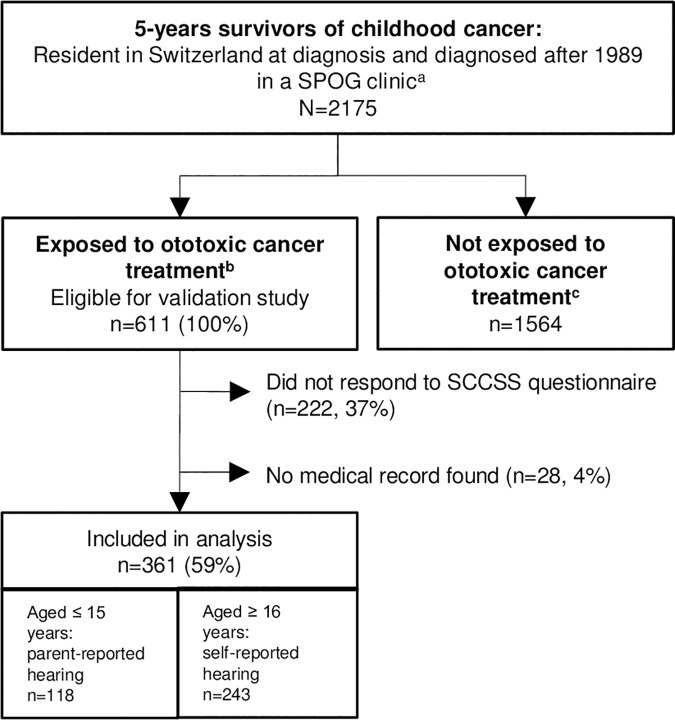
Flow chart of study population. Abbreviations: SPOG, Swiss Pediatric Oncology Group; SCCSS, Swiss Childhood Cancer Survivor Study.^a^SPOG clinics including the following clinics with paediatric oncology units: Kantonsspital Aarau AG, Universitäts-Kinderspital Basel, Ospedale S. Giovanni Bellinzona, Universitäts-Kinderklinik Bern, Hospital des Enfants Geneve, CHUV Lausanne, Kantonsspital Luzern, Ostschweizer Kinderspital St. Gallen, Universitäts-Kinderspital Zürich. ^b^Includes survivors who have received platinum compounds or cranial radiation.^c^Includes survivors who have not received platinum compounds or cranial radiation.

We included 2175 survivors in this validation study who were diagnosed in a Swiss specialized pediatric oncology (SPOG) clinic after 1989 because medical records of patients diagnosed earlier were rarely available (Fig 1). To determine whether they had been exposed to ototoxic cancer treatment, we obtained treatment-related information from the SCCR that included type and year of cancer diagnosis, and age at cancer diagnosis, chemotherapy, radiotherapy, surgery, and bone marrow transplantation (BMT). Cancer diagnoses were classified according to the International Classification of Childhood Cancer, third edition (ICCC-3) [14].

### Assessment of hearing

#### The SCCSS questionnaire survey

Between 2007 and 2013, we traced all addresses of survivors and sent them a long questionnaire. Parents of survivors ≤15 years old completed the questionnaire for their children. Survivors who were ≥16 completed questionnaires themselves. Nonresponders received a second copy of the questionnaire. If they again did not respond, we contacted them by phone. The main topics covered by the questionnaire were quality of life, somatic health, use of current medication and health services, psychological distress, health behaviors, and socioeconomic status. The detailed study design has been published elsewhere [12]. The SCCSS questionnaire included questions on hearing (S1 Fig). We asked the participant (or participant’s parent) whether she/he had ever been told by a doctor that she/he had problems with hearing. If the answer was yes, the participant was asked to indicate the laterality (unilateral/bilateral) of the problem, and to identify its severity by selecting one of the three ratings: mild problems, not requiring hearing aids or completely corrected with hearing aid; moderate problems, not completely corrected by hearing aid; or severe problems, not correctable by hearing aid (deaf). We categorized severity of questionnaire-reported hearing loss as mild, moderate, or severe and created also a binary variable (yes/no) for questionnaire-reported hearing loss that combined those with mild, moderate or severe hearing loss in one category and those with normal hearing to another category.

#### Medical records

**Fig 2:**
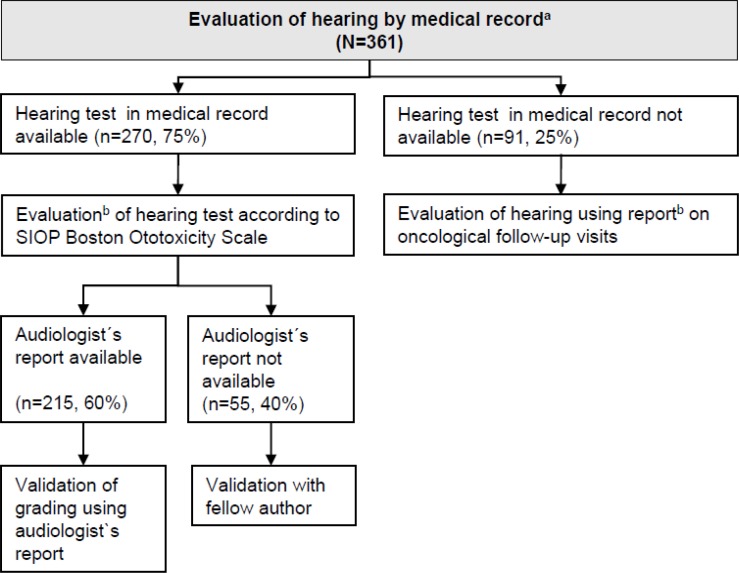
Flow chart on data collection via medical records. Abbreviations: SIOP, International Society of Pediatric Oncology; ENT, Ear-nose-throat. ^a^We collected information from medical records of the paediatric oncological departments and ENT departments. ^b^When we found more than one report or hearing test with differing results, we used the most recent report/test.

In 2015 we collected hearing tests, the corresponding audiologists´ reports, and oncological follow-up reports from the original medical records in the clinic archives of ear-nose-throat (ENT) departments and pediatric oncology clinics. When we found multiple reports or tests with hearing results that differed, we used the most recent result. We only considered reports or tests that were dated before the completion of the SCCSS questionnaire. The first author (A.W.) graded all hearing tests from these records for each ear separately and for frequencies up to 8 kHz according to the SIOP Boston Ototoxicity Scale (Fig 2) [15]. A.W. was blinded to the hearing outcome measured by the questionnaire. Unclear cases were discussed with a senior audiologist (M.K.). When hearing tests were accompanied by the reports of audiologists, A.W. used the audiologist results in a second step to validate her grading. When we found only oncological follow-up reports, we looked for information on hearing in medical histories and examinations. If no information on hearing was available in the oncological follow-up reports, we assumed normal hearing and assigned grade 0 (≤20 dB) of the SIOP Boston Ototoxicity Scale. To justify the use of oncological follow-up reports only for validation purposes, we checked the reports of 45 randomly selected persons with hearing loss according to hearing tests to see if the results of the hearing test were mentioned in the corresponding oncological follow-up report. In 41 out of 45 (91%) cases, the pathological result of the hearing test was mentioned in the oncological report. This indicates that hearing test results were forwarded to the pediatric oncology departments and recorded in oncological follow-up reports as part of clinical standard procedures. We defined hearing loss from medical records as ≥grade 1 (>20 dB above 4kHz) according to the SIOP Boston Ototoxicity scale in the most affected ear. We graded severity of hearing loss from medical records from 0 to 4 [15]. We also noted the date of the follow-up used for grading of hearing and categorized the interval between that follow-up and questionnaire into four categories: 0–2, 3–4, 5–9, and 10–17 years.

### Statistical analysis

First, we determined prevalence, severity, and laterality of hearing loss assessed by questionnaire and by medical records, and compared differences in prevalence using McNemar statistics.

Second, we assessed agreement between questionnaires and medical records. We calculated percent agreement (proportion of those with agreement in questionnaire and medical records) and Cohen´s kappa statistics [16]. Kappa value >0.75 was considered as an excellent agreement beyond chance, a kappa of 0.40–0.75 as intermediate to good agreement, and a kappa below 0.40 as poor agreement [17]. To investigate whether agreement varies between subgroups, we stratified measures by questionnaire respondent, sociodemographic characteristics, and time between follow-up and questionnaire. We used chi-square statistics to compare percent agreement between subgroups.

Third, we evaluated validity of questionnaire-reported hearing. Information from medical records was treated as reference. We calculated sensitivity, specificity, positive predictive value (PPV), and negative predictive value (NPV). Sensitivity was the proportion of those with hearing loss as defined by the medical records whom the questionnaire classified correctly. Specificity was the proportion of those with normal hearing whom the questionnaire classified correctly. PPV was the proportion of those with questionnaire-reported hearing loss, in whom hearing loss was confirmed by medical records. NPV was the proportion of those with questionnaire-reported normal hearing, in whom normal hearing was confirmed by medical records. To investigate how validity might vary between subgroups, we also stratified measures of validity.

Last, we assessed whether the questionnaire assessed hearing loss equally well for different severity levels of hearing loss. We calculated sensitivity and PPV stratified by severity level and laterality of hearing loss.

We also performed a sensitivity analysis to compare results from all respondents (n = 361) to results from respondents for whom we found original hearing tests in medical records (n = 270) by excluding those for whom we did not find hearing tests in medical records and only used information from oncological follow-up (n = 91).

We used Stata (Version 13, Stata Corporation, Austin, Texas) for all analyses.

## Results

### Characteristics of study population

Of the 2175 survivors diagnosed after 1989 in a SPOG clinic, 611 (28%) had received ototoxic cancer treatment. Among these, 222 did not reply to the questionnaire and 28 had missing medical records (Fig 1). This resulted in 361 persons (59% of those who received otoxic treatment) who were available for analyses. There were no significant differences between those who were included in the analysis and those who were not, except for a slight difference in type of cancer diagnosis (p = 0.028, Table 1). Fifty-four percent of the study participants were male. Median (interquartile range) age at survey was 18 (6–30) years and median age at diagnosis 5 (0–15) years. The most common diagnosis was CNS tumor (27%), followed by leukemia (24%), and bone tumor (10%). Of those who had received chemotherapy, 22% had been treated with carboplatin, 26% with cisplatin, 20% with both carboplatin and cisplatin, and 32% with neither agent. Of those who had received radiation, 91% had received cranial radiation. Of those who had undergone surgery, 38% had brain surgery. Eleven percent had BMT. Median time between last follow-up and questionnaire was 5 (2–7) years.

**Table 1:** Characteristics of study population.

	Eligible population included in the study N = 361		Eligible population not included in the study N = 250		
Sociodemographic characteristics	n	(%)	n	(%)	P^a^
**Gender**					0.650
Female	167	(46)	111	(44)	
Male	194	(54)	139	(56)	
**Age at survey**					0.296
5–15 years	118	(33)	67	(27)	
16–20 years	109	(30)	83	(33)	
21–40 years	134	(37)	100	(40)	
**Migration background**^**b**^					
No	288	(80)	n.a.^d^		
Yes	73	(20)			
**Education**^**c**^					
Primary education	37	(12)	n.a.^d^		
Secondary education	216	(60)			
Tertiary education	100	(28)			
**Clinical characteristics**					
**Diagnosis (ICCC3)**					0.028
I Leukemia	88	(24)	65	(26)	
II Lymphoma	13	(4)	15	(6)	
III CNS tumor	99	(27)	59	(24)	
IV Neuroblastoma	31	(9)	21	(8)	
V Retinoblastoma	28	(8)	10	(4)	
VI Renal tumor	3	(1)	12	(5)	
VII Hepatic tumor	12	(3)	5	(2)	
VIII Bone tumor	35	(10)	17	(7)	
IX Soft tissue sarcoma	29	(8)	22	(9)	
X Germ cell tumor	22	(6)	21	(8)	
XI &XII Other rare tumor	1	(0)	3	(1)	
**Treatment**					
**Chemotherapy**	341	(94)	230	(92)	0.227
*No platinum chemotherapy*	*108*	*(32)*	*92*	*(40)*	
*Carboplatin*	*76*	*(22)*	*48*	*(21)*	
*Cisplatin*	*89*	*(26)*	*45*	*(20)*	
*Cisplatin and Carboplatin*	*68*	*(20)*	*45*	*(20)*	
**Radiotherapy**	228	(63)	169	(68)	0.314
*No cranial radiation*	*21*	*(9)*	*21*	*(12)*	
*Cranial radiation <30 Gy*	*87*	*(38)*	*70*	*(41)*	
*Cranial radiation 30–49 Gy*	*25*	*(11)*	*15*	*(9)*	
*Cranial radiation ≥ 50 Gy*	*87*	*(38)*	*57*	*(34)*	
*Unknown*	*8*	*(4)*	*6*	*(4)*	
**Surgery**	252	(70)	169	(70)	0.905
*Brain surgery*	*95*	*(38)*	*60*	*(36)*	.
**Bone marrow transplantation**	40	(11)	28	(11)	0.284
**Time between last follow-up and questionnaire**					
0–2 years	135	(37)	n.a.^d^		
3–4 years	57	(16)			
5–9 years	125	(35)			
10–17 years	41	(11)			
Unknown	3	(1)			

Abbreviations: N, number; n.a., not applicable; ICCC3, International Classification of Childhood Cancer—Third edition; CNS, central nervous system; Gy, Gray.

^a^p-values calculated from chi-square statistics comparing included to excluded survivors.

^b^Survivors were coded as having a migration background if they were not born in Switzerland, had no Swiss citizenship at birth or had at least one parent without Swiss citizenship.

^c^Survivor´s and parent´s education was classified into three categories: primary education (compulsory schooling only [≤9 years]), secondary education (vocational training [10–13 years], higher vocational training or college), or tertiary education (university or technical college education). If the father and mother had achieved different levels of education, we selected the parent with the highest education. Education includes parental education when parents answered the questionnaire, and survivors´ education when survivors answered the questionnaire.

^d^Migration background, education, and time between follow-up and questionnaire are not available for nonresponding parents and survivors.

### Assessment of hearing by questionnaire and medical records

For 118 of the 361 respondents (33%) parents answered the questionnaire because they were younger than 16 (parent-reported). The 243 (67%) older respondents answered the questionnaire themselves (survivor-reported, Fig 1). Hearing tests were found in medical records of 270 respondents (75%) (Fig 2). Among those, 215 (60%) also had an audiological report. The report confirmed hearing loss at or above grade 1 in 207 of these 215 cases (96%). The remaining 8 (4%) had high-frequency hearing loss according to hearing tests (grade 1, >20 dB HL above 4kHz) that was not defined as hearing loss by audiologist/clinician. For 91 of the respondents (25%), we used oncological follow-up reports to assess hearing because no hearing test was available in medical records.

### Prevalence and severity of hearing loss

Prevalence of hearing loss was nearly the same in the questionnaire reports and medical records (27% vs. 31%, p = 0.040; Table 2). Most respondents reported mild (16%) or moderate hearing loss (9%), and only few had severe hearing loss (2%). In the medical records, severity grades of hearing loss according to SIOP Boston Ototoxicity Scale were 6% with grade 1, 9% with grade 2, 7% with grade 3, and 8% with grade 4. Seven percent of respondents reported unilateral hearing loss, 17% bilateral hearing loss, and 3% did not specify. Laterality of hearing loss assessed by medical records resulted in 6% with unilateral and 25% with bilateral hearing loss.

**Table 2:** Prevalence, severity, and laterality of hearing loss assessed by questionnaire or medical records.

	N (%)
**Hearing measured by questionnaire**	
Normal hearing	265 (73)
Hearing loss	96 (27)
Severity	
Mild hearing loss	59 (16)
Moderate hearing loss	31 (9)
Severe hearing loss (deafness)	6 (2)
Laterality	
Unilateral	24 (7)
Bilateral	63 (17)
Unknown	9 (3)
**Hearing measured by medical records**	
Normal hearing^a^	249 (69)
Hearing loss	112 (31)
Severity^b^	
Grade 1 (>20 dB HL above 4kHz)	23 (6)
Grade 2 (>20 dB HL at 4kHz and above)	34 (9)
Grade 3 (>20 dB HL at 2kHz and above)	25 (7)
Grade 4 (>40 dB HL at 2kHz and above)	30 (8)
Laterality	
Unilateral	21 (6)
Bilateral	91 (25)

Abbreviations: N, number; SIOP, International Society of Pediatric Oncology.

^a^Includes SIOP Boston Ototoxicity Scale Grade 0.

^b^According to SIOP Boston Ototoxicity Scale

### Agreement and validity

Overall, agreement between questionnaires and medical records was good (85%, 95% CI 81–89%; kappa 0.64, 95% CI 0.55–0.72; Table 3). Results differed neither by type of respondent (survivor- or parent-reported) nor by sociodemographic characteristics (gender, education, migration) (Table 3). Agreement was more better when the time interval between follow-up and questionnaire was shorter: up to 87% for intervals up to 9 years, and 71% for intervals of 10–17 years (p = 0.051). Questionnaire-reported hearing loss had a sensitivity of 69% (95% CI 59–77%), and a specificity of 92% (95% CI 88–95%) compared to medical records (Table 3). Among those who reported hearing loss, medical records confirmed hearing loss in 80% (95% CI 71–88%). Among those who reported normal hearing, medical records confirmed normal hearing in 87% (95% CI 82–91%).

**Table 3:** Measures of agreement and validity for questionnaire-reported hearing.

Hearing loss by medical record/questionnaire					Agreement			Validity			
	Y/Y^a^	N/Y^b^	Y/N^c^	N/N^d^	Percent agreement, %	Kappa	p^e^	Sensitivity^f^, %	Specificity^f^, %	PPV^f^, %	NPV^f^, %
Overall	77	19	35	230	85 [81–89]	0.64 [0.55–0.72]		69 [59–77]	92 [88–95]	80 [71–88]	87 [82–91]
By type of questionnaire report													
Parent-reported	25	5	9	79	88 [81–93]	0.70 [0.56–0.85]	0.251	74 [57–87]	94 [87–98]	83 [65–94]	90 [82–95]
Survivor-reported	52	14	26	151	84 [78–88]	0.60 [0.50–0.72]		67 [55–77]	92 [86–95]	79 [67–88]	85 [79–90]
By gender										
Female	35	9	11	112	88 [82–93]	0.70 [0.57–0.82]	0.140	76 [61–87]	93 [86–97]	80 [65–90]	91 [85–96]
Male	42	10	24	118	82 [76–88]	0.59 [0.47–0.71]		64 [51–75]	92 [86–96]	81 [68–90]	83 [76–89]
By education											
Primary education	11	3	6	23	79 [64–90]	0.55 [0.29–0.81]	0.472	65 [38–85]	89 [70–98]	79 [49–95]	79 [60–92]
Secondary education	45	11	21	139	85 [80–90]	0.64 [0.52–0.75]		68 [56–79]	93 [87–96]	80 [68–90]	87 [81–92]
Tertiary education	20	5	8	67	87 [79–93]	0.67 [0.50–0.83]		71 [51–87]	93 [85–98]	80 [59–93]	89 [80–95]
By migration background											
No	64	17	28	179	84 [80–88]	0.63 [0.53–0.73]	0.481	70 [59–79]	91 [87–95]	79 [69–87]	87 [81–91]
Yes	13	2	7	51	88 [78–94]	0.66 [0.46–0.86]		65 [41–85]	96 [87–100]	87 [60–98]	88 [77–95]
By time between follow-up and questionnaire											
0–2 years	33	9	8	85	87 [81–92]	0.70 [0.57–0.84]	0.051	81 [65–91]	90 [83–96]	79 [63–90]	91 [84–96]
3–4 years	12	3	5	37	86 [74–94]	0.65 [0.43–0.87]		71 [44–90]	93 [80–98]	80 [52–96]	88 [74–96]
5–9 years	23	5	11	86	87 [80–93]	0.66 [0.50–0.81]		68 [50–83]	95 [88–98]	82 [63–94]	89 [81–94]
10–17 years	9	1	11	20	71 [54–84]	0.41 [0.16–0.65]		45 [23–69]	95 [76–100]	90 [56–100]	65 [45–81]

Abbreviations: n.a., not applicable.

^a^Y/Y, hearing loss in medical record and hearing loss in questionnaire.

^b^N/Y, normal hearing in medical record and hearing loss in questionnaire.

^c^Y/N, hearing loss in medical record and normal hearing in questionnaire.

^d^N/N, normal hearing in medical record and normal hearing in questionnaire.

^e^p-values calculated from chi-square statistic comparing percent agreement by strata.

^f^Data from medical records were considered as reference.

#### Severity and laterality of hearing loss

Sensitivity of questionnaire reports varied with different levels of hearing loss (Table 4, S2 and S3 Figs). Sensitivity was highest in respondents with moderate (grade 2) and severe (grade 3–4) hearing loss according to the SIOP Boston Ototoxicity Scale: 71%, 95% CI 53–85%; and 86%, 95% CI 73–94% respectively. Sensitivity was lowest in those with mild (grade 1) hearing loss: 26%, 95% CI 10–48%. After stratification for laterality of hearing loss, we found that at all levels of severity those with unilateral hearing loss more often under-report their impairment than those with bilateral hearing loss: grade 1, 10% vs. 39%; grade 2, 33% vs. 74%; grades 3–4, 50% vs. 92%.

**Table 4:** Measures of validity for questionnaire-reported hearing for different degrees of hearing loss.

Hearing loss according to medical records	Sensitivity^a^, %	PPV^a^, %
Mild including grade 1^b^	26 [10–48]	24 [9–45]
Unilateral	10 [1–45]	5 [1–25]
Bilateral	39 [14–68]	21 [7–42]
Moderate including grade 2^b^	71 [53–85]	56 [40–71]
Unilateral	33 [1–91]	5 [1–25]
Bilateral	74 [55–88]	55 [39–70]
Severe including grade 3–4^b^	86 [73–94]	71 [59–82]
Unilateral	50 [16–84]	17 [5–39]
Bilateral	92 [80–98]	69 [56–80]

Abbreviation: PPV, Positive predictive value.

^a^Data from medical records were considered as reference.

^b^Severity grades according to SIOP Boston Ototoxicity Scale.

### Sensitivity analysis

We found no important differences in the sensitivity analyses that compared results of all data to results of data excluding those for whom we did not find hearing tests in medical records and only used information from oncological follow-up (n = 91) (S1 and S2 Tables). In this analysis, agreement between questionnaires and medical records was 83% (95% CI 78–87%) and kappa 0.63 (95% CI 0.54–0.73). Questionnaires had a sensitivity of 69% (95% CI 59–77%), a specificity of 92% (95% CI 87–96%), a PPV of 86% (95% CI 78–93%) and NPV of 81% (95% CI 74–86%) compared to medical records (S1 Table).”

## Discussion

### Principal findings

In this study, the first to comprehensively investigate agreement and validity of questionnaire-reported hearing in childhood cancer survivors, we found that questionnaires assessed hearing in childhood cancer survivors with acceptable validity. Medical records agreed with 85% of respondents’ questionnaires, which correctly assessed normal hearing in 92% of those with normal hearing and hearing loss in 69% of those with hearing loss. Results did not differ by questionnaire respondent (survivor- or parent-report) or sociodemographic characteristics. Questionnaires did underestimate mild or unilateral hearing loss.

### Limitations and strengths

We assumed that medical records were complete and correct. However, we would have missed tests of survivors who had their hearing tested after oncological long-term follow-up at a private practice. We also used information on hearing from the oncological follow-up reports, when we found no hearing assessment in the medical records and assumed normal hearing when we found no information on hearing in those reports. This could have contribute to discrepancies in hearing loss between questionnaires and medical records, and may have increased the number of cases with false positive results. However, the sensitivity analysis suggested that this has not led to a relevant distortion of the results. Respondents with a long period between last follow-up and questionnaire might recall the doctor´s diagnosis less well, but validity decreased only in those with more than 10 years between follow-up and questionnaire. Only a minority of the respondents (11%) had such a long period. Finally, our results cannot be extrapolated to children who have not received ototoxic cancer treatment.

Our study is strengthened by a study population that is large compared to those of previous validation studies of childhood cancer survivors [18–20]. Because respondents received in many cases comprehensive audiological follow-up, we could ask them about the results of clinical assessment of their hearing status (S1 Fig) rather than only being able to rely upon subjective perception of their own hearing—as is often the case in validation studies in the general population [7–10].

### Comparison with other studies

This is the first study to compare the questionnaire-reported prevalence of hearing loss with medical records in one group of childhood cancer survivors. The prevalence of hearing loss was slightly lower assessed by questionnaire than by medical records (27% vs. 31%). The prevalence of hearing loss is hard to compare in different study populations because of differences in diagnostic groups, cancer treatment, and type of auditory grading. In previous studies the prevalence of questionnaire-reported hearing loss in groups at risk of ototoxicity ranged from 16 to 31% [2,4,21], and was even higher when prevalence was assessed by retrospective data collection in medical records (12–90%) [3,22–24]. However, previous studies that are similar to ours may have underestimated the prevalence of hearing loss just as our study did for mild and unilateral hearing loss.

No other study has investigated validity and agreement of questionnaire-reported hearing with medical records in childhood cancer survivors. Most publications on validity of questionnaire-reported hearing studied populations of older age using questions on subjective perception of hearing such as “Do you feel you have hearing loss?” [8,9,25–29]. In an Australian study that pooled seven data sets with 23,001 respondents asked for self-assessment of hearing (age 45–103 years), sensitivity ranged from 62–78% and specificity from 41–85% [29]. Our question “Have you ever been told by a doctor that you have, or have had hearing loss?” performed similarly in those with hearing loss (sensitivity 69%), but better in those with normal hearing (specificity 92%).

We found very similar results for agreement and validity when either survivors or parents reported on hearing. Both survivors and parents recall hearing equally well. There is no other study investigating the difference between survivor- and parent-reported hearing.

Validation studies on hearing in mainly elderly people reported that younger, male, and more highly educated respondents report hearing more accurately [8,9,26,29]. Our findings and those of other validation studies of childhood cancer survivors that also asked survivors for health problems reported by a clinician suggested no effect of sociodemographic factors [18,20]. The different types of questions might be the cause for the discrepancy between findings from the general population and childhood cancer survivors as self-perception might be more affected by age, gender, or education level.

Not surprisingly, we found that survivors with mild and unilateral hearing loss are more likely to under-report hearing loss than survivors with severe hearing loss. This may have two causes. First, survivors may not have been aware of their mild hearing loss because their audiologist/clinician did not tell them about it. In 4% of cases, which we validated with the audiologist´s results of hearing tests, audiologists did not report on hearing loss though the audiogram showed limited results in the high frequencies. Second, unlike severe hearing loss unilateral or mild hearing loss may affect daily life little or not at all and fewer survivors may recall such a diagnosis, especially when it was some years ago. Several studies have also reported that the accuracy of questionnaire reports of mild hearing loss is low among the elderly [7,9,27]. They have shown that single questions such as “Do you have a problem with your hearing?” are less able to detect mild hearing loss than more comprehensive instruments like the Hearing Handicap Inventory for the Elderly—Screening Version (HHIE-S) [30]. A recent systematic review that described patient-reported measures for hearing loss identified only two instruments, the Health Utilities Index (HUI) [31] and the Hearing Measurement Scale (HMS) [32] that are used with childhood cancer patients [33]. The HUI includes six questions, the HMS 44 questions, and both return hearing-specific summary scores. Researchers reported that none of the questionnaires appeared ideal based upon the number of questions, and wording for children or adolescents, and psychometric properties were never tested in this population [33].

### Interpretation of results

Studies of hearing loss after treatment of childhood cancer ideally should collect data from medical records or, to obtain the most valid results, prospectively perform audiometry assessments. However, these approaches are often not feasible because they are expensive, and access to full medical records or additional audiometry assessments may be complicated. Many studies therefore assessed hearing loss by questionnaires, which inquire about doctor-diagnosed hearing problems. Reports of survivors and their parents appear equally valid.

Questionnaires assess moderate or severe hearing loss well, but underestimate mild and unilateral hearing loss. Our questionnaire missed 90% of cases with mild unilateral and 67% of cases with moderate unilateral hearing loss. Particularly in the absence of well established questionnaires we suggest that researchers validate questionnaire-reported data in at least a subsample to avoid misclassification and reduce bias. Since level of hearing loss differentially affects the accuracy of hearing reports, the results observed for effects of ototoxic risk factors on hearing or the impact of hearing loss on an outcome could be biased. Instruments currently used in clinical assessment of hearing loss in childhood cancer patients such as the HUI and the HMS have questions that focus on difficulties following group conversations with or without a noisy environment. In a future project, we will investigate asking about this type of hearing difficulty to assess mild or unilateral hearing loss independent of clinicians who may not measure or report mild hearing loss.

### Conclusion

Questionnaires are a useful tool to assess hearing in childhood cancer survivors. However, they underestimate mild and unilateral hearing loss, so researchers should investigate whether inquiring about difficulty following conversation in a noisy environment can improve the assessment of this type of hearing loss in childhood cancer survivors.

## Supporting information

S1 Fig Questions on hearing from the questionnaire for adults and adolescents of the Swiss Childhood Cancer Survivor Study, translated to English. (TIF)

S2 Fig Sensitivity of questionnaire-reports by laterality and severity of hearing loss. Abbreviation: HL, hearing loss.
* according to medical records.
(TIF)

S3 Fig Questionnaire-reported severity of hearing loss stratified by severity of hearing loss assessed by medical records. Abbreviation: HL, hearing loss.
*according to SIOP Boston Ototoxicity Scale.
(TIF)

S1 Table Measures of agreement and validity for questionnaire-reported hearing–Sensitivity analysis including survivors with hearing test (n = 270). (PDF)

S2 Table Measures of validity for questionnaire-reported hearing for different degrees of hearing loss–Sensitivity analysis including survivors with hearing test (n = 270). (PDF)
