# Supplementary figures and images for: Validation of questionnaire-reported hearing with medical records: A report from the Swiss Childhood Cancer Survivor Study
Source: PLoS One. 2017 Mar 23;12(3):e0174479. doi: 10.1371/journal.pone.0174479 (PMC5363962; doi:10.1371/journal.pone.0174479)

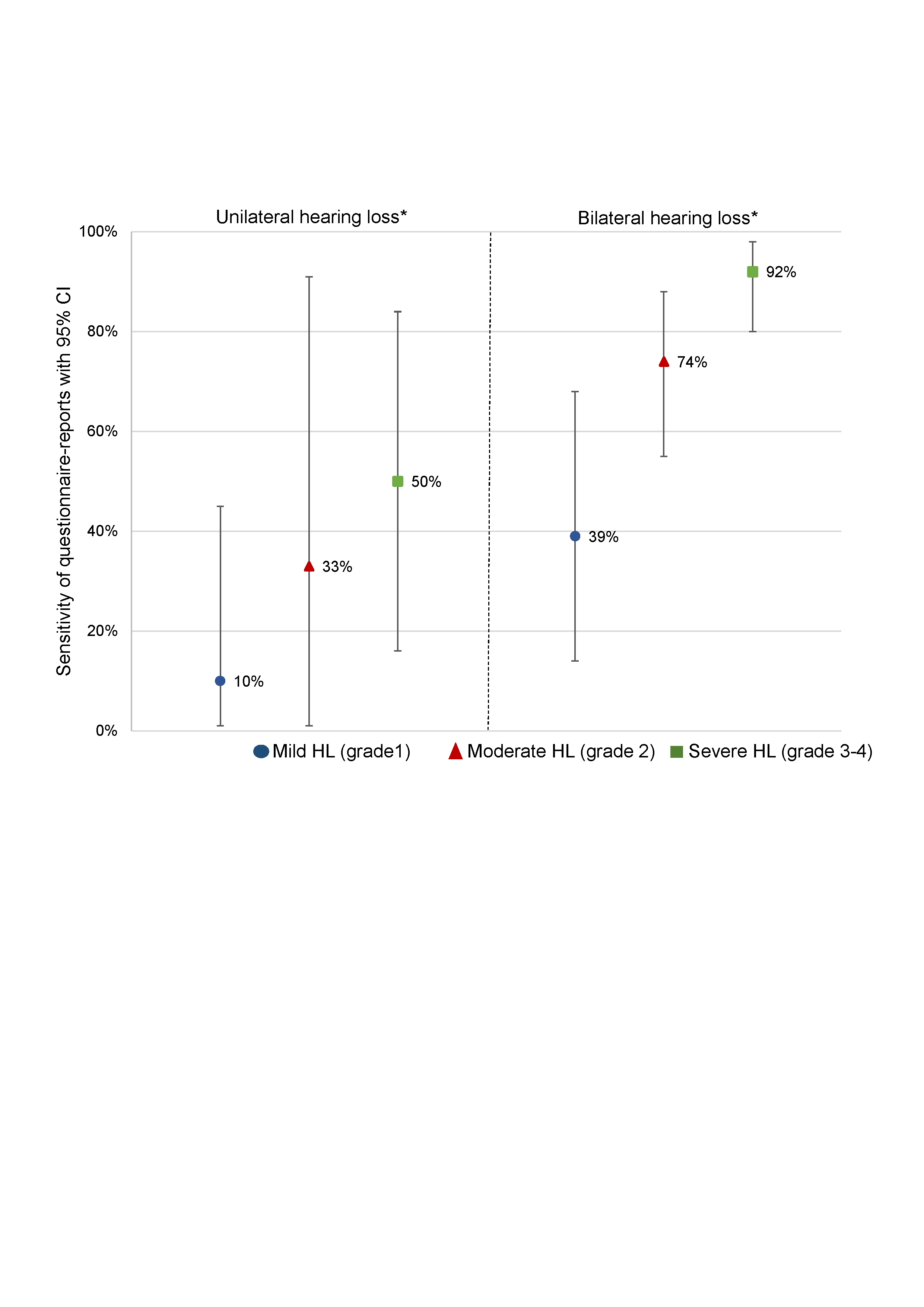

Supplement: S2 Fig — Abbreviation: HL, hearing loss. * according to medical records. (TIF) [file pone.0174479.s002.tif]

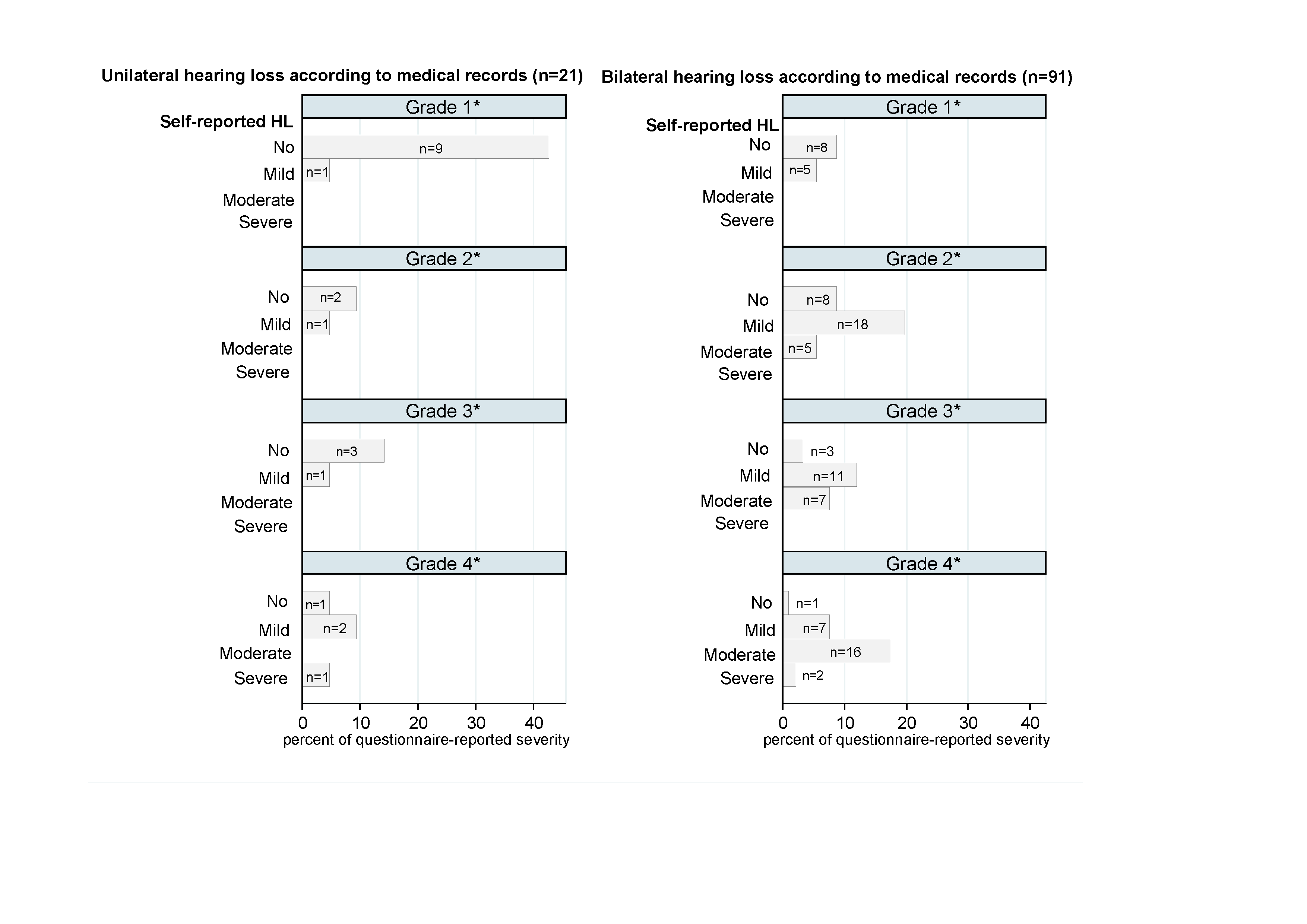

Supplement: S3 Fig — Abbreviation: HL, hearing loss. *according to SIOP Boston Ototoxicity Scale. (TIF) [file pone.0174479.s003.tif]
