# Supplementary material for: Validation of questionnaire-reported hearing with medical records: A report from the Swiss Childhood Cancer Survivor Study
Source: PLoS One. 2017 Mar 23;12(3):e0174479. doi: 10.1371/journal.pone.0174479 (PMC5363962; doi:10.1371/journal.pone.0174479)
Supplement: S1 Table — (PDF) [file pone.0174479.s004.pdf]

S4 Table. Measures of agreement and validity for questionnaire-reported hearing – Sensitivity analysis including survivors with hearing test (n=270)

Hearing loss by medical record/questionnaire					Agreement			Validity			
	Y/Y[a]	N/Y[b]	Y/N[c]	N/N[d]	Percent agreement, %	Kappa	p[e]	Sensitivity[f], %	Specificity[f], %	PPV[f], %	NPV[f], %
**Overall**	77	12	35	146	83 [78-87]	0.63 [0.54-0.73]		69 [59-77]	92 [87-96]	86 [78-93]	81 [74-86]
By type of questionnaire report											
Parent-reported	25	5	9	61	86 [78-92]	0.68 [0.53-0.83]	0.339	74 [56-87]	92 [83-97]	83 [65-94]	87 [77-94]
Self-reported	52	7	26	85	81 [74-86]	0.60 [0.48-0.72]		67 [55-77]	92 [85-97]	88 [77-95]	77 [68-84]
By gender											
Female	35	6	11	70	86 [79-92]	0.70 [0.56-0.83]	0.250	76 [61-87]	92 [84-97]	85 [71-94]	86 [77-93]
Male	42	6	24	76	80 [72-86]	0.58 [0.45-0.71]		64 [51-75]	93 [85-97]	88 [75-95]	76 [66-84]
By education											
Primary education	11	1	6	14	78 [60-91]	0.57 [0.30-0.84]	0.850	65 [38-86]	93 [68-100]	92 [61-100]	70 [46-88]
Secondary	45	7	21	91	82 [76-88]	0.63 [0.51-0.75]		68 [56-79]	93 [86-97]	86 [74-94]	81 [73-88]
Tertiary education	20	4	8	40	83 [73-91]	0.64 [0.46-0.82]		71 [51-87]	91 [78-97]	83 [63-95]	83 [70-93]
By migration background											
No	64	11	28	114	82 [76-87]	0.62 [0.52-0.73]	0.675	70 [59-79]	91 [85-96]	85 [75-92]	80 [83-86]
Yes	13	1	7	32	85 [72-93]	0.66 [0.45-0.87]		65 [41-85]	97 [84-100]	93 [66-100]	82 [66-92]
By time between follow-up and questionnaire											
0-2 years	33	6	8	47	85 [76-92]	0.70 [0.55-0.84]	0.127	80 [65-91]	89 [77-96]	85 [69-94]	85 [73-94]
3-4 years	12	3	5	25	82 [68-92]	0.61 [0.37-0.85]		71 [44-90]	89 [72-98]	80 [52-96]	83 [65-94]
5-9 years	23	2	11	61	87 [78-93]	0.69 [0.53-0.84]		68 [49-83]	97 [89-100]	92 [74-99]	85 [74-92]
10-17 years	9	1	11	13	65 [46-80]	0.34 [0.09-0.60]		45 [23-68]	93 [66-100]	90 [56-100]	54 [33-74]

Abbreviations: n.a., not applicable.
[a]Y/Y, hearing loss in medical record and hearing loss in questionnaire.
[b]N/Y, normal hearing in medical record and hearing loss in questionnaire.
[c]Y/N, hearing loss in medical record and normal hearing in questionnaire.
[d]N/N, normal hearing in medical record and normal hearing in questionnaire.
[e]p-values calculated from chi-square statistic comparing percent agreement by strata.
[f]Data from medical records were considered as reference.
