# Supplementary material for: Validation of questionnaire-reported hearing with medical records: A report from the Swiss Childhood Cancer Survivor Study
Source: PLoS One. 2017 Mar 23;12(3):e0174479. doi: 10.1371/journal.pone.0174479 (PMC5363962; doi:10.1371/journal.pone.0174479)
Supplement: S2 Table — (PDF) [file pone.0174479.s005.pdf]

S5 Table. Measures of validity for questionnaire-reported hearing for different degrees of hearing loss – Sensitivity analysis including survivors with hearing test (n=270)

Hearing loss according medical records	Sensitivity[a], %	PPV[a], %
Mild including grade 1[b]	26 [10-48]	33 [13-59]
Unilateral	10 [0-45]	8 [0-36]
Bilateral	38 [14-68]	29 [10-56]
Moderate including grade 2[b]	71 [53-85]	67 [49-81]
Unilateral	33 [1-91]	8 [0-36]
Bilateral	74 [55-88]	66 [48-81]
Severe including grade 3-4[b]	85 [73-94]	80 [67-89]
Unilateral	50 [16-84]	25 [7-52]
Bilateral	91 [80-98]	78 [65-88]

Abbreviation: PPV, Positive predictive value; NPV, Negative predictive value.
[a]Data from medical records were considered as reference.
[b]Severity grades according to SIOP Boston Ototoxicity Scale
